# Non-motor symptoms as critical predictors of quality of life in Parkinson’s disease: a machine learning approach

**DOI:** 10.1186/s12955-025-02451-2

**Published:** 2025-12-06

**Authors:** Daniel Magano, António S. Barros, João Massano, Laila Alsuwaidi, Tiago Taveira-Gomes

**Affiliations:** 1 Ph.D. Program in Health Data Science, Faculty of Medicine, University of Porto, Porto, 4200-319 Portugal; 2 Medical Department, BIAL-Portela & Cª., S.A, São Mamede do Coronado, 4745-457 Portugal; 3 RISE-Health, Department of Surgery and Physiology, Faculty of Medicine, University of Porto, Porto, 4200-319 Portugal; 4 Department of Clinical Neurosciences and Mental Health, Faculty of Medicine, University of Porto, Porto, 4200-319 Portugal; 5 Department of Neurology, Centro Hospitalar Universitário de São João, Porto, 4200-319 Portugal; 6 Department of Community Medicine, Information and Decision in Health, Faculty of Medicine, University of Porto, Porto, 4200-319 Portugal; 7 Faculty of Health Sciences, University Fernando Pessoa, Porto, 4200-150 Portugal; 8 SIGIL Scientific Enterprises, Dubai, 4076 United Arab Emirates

**Keywords:** Parkinson’s disease, Health-related quality of life, Predictive modeling, Machine learning, Parkinson’s disease questionnaire-39, Explainable machine learning

## Abstract

**Background:**

Parkinson’s disease (PD) considerably impacts health-related quality of life (HRQoL) through motor and non-motor symptoms. The Parkinson’s Disease Questionnaire-39 (PDQ-39) is the most widely used tool to assess HRQoL, encompassing eight dimensions and a Summary Index providing an overall score. Despite advances in machine learning (ML) for predicting disease symptoms and progression, its application to predict HRQoL across these dimensions remains underexplored.

**Methods:**

This study uses complete-case data for 478 of 861 patients from PRISM, a cross-sectional observational survey conducted in six European countries in 2018–2019. Participants were adults with PD recruited through advocacy groups and clinical centers who completed online assessments, providing data on demographics, medication, comorbidities, and disease characteristics (Tolosa et al., 2021). ML models were trained to predict PDQ-39 dimensions and Summary Index scores (0–100; higher = worse HRQoL). Features were preselected using the Boruta algorithm on the training data. Model selection was based on the lowest mean RMSE from 100 bootstrap resamples on the training set. Selected models were then retrained using 1000 bootstrap resamples for robust performance estimation. Final performance was evaluated on a held-out 20% validation set using R², MAE, and RMSE. Feature importance was assessed using permutation importance with MAE loss (100 permutations) on the held-out validation set. Factor Analysis of Mixed Data (FAMD) was used to explore patterns between non-motor symptoms and PDQ-39.

**Results:**

Selected models: xgbTree (Summary Index; Activities of Daily Living) and gaussprPoly (all other PDQ-39 dimensions). On the validation set, Summary Index/ Cognitions showed the strongest performance with R² = 0.56/0.53, MAE = 9.60/12.39, RMSE = 12.66/16.20. Permutation feature importance ranked the Non-Motor Symptoms Questionnaire score (sum of 30 non-motor symptoms, range 0–30) as the most important predictor across all models. FAMD showed clustering of Social Support, Bodily Discomfort, and Stigma dimensions with Anxiety.

**Conclusions:**

Our findings demonstrate the critical role of non-motor symptoms in predicting HRQoL in patients with PD. While ML models effectively predict overall HRQoL and cognitive aspects, achieving comparable performance on other dimensions may require additional variables to reduce error. These insights emphasize comprehensive treatment strategies addressing both motor and non-motor symptoms.

## Background

Parkinson’s disease (PD) is a progressive neurodegenerative disorder that affects millions of people worldwide, with its global prevalence more than doubling between 1990 and 2015, highlighting the growing burden of this condition [1, 2]. PD is primarily characterized by motor symptoms such as resting tremor, rigidity, bradykinesia, and postural instability, which are often accompanied by a variety of non-motor symptoms (NMS), including cognitive impairment, mental health disorders, sleep disturbances, pain, and sensory disturbances [2–4]. These symptoms considerably reduce the health-related quality of life (HRQoL) in individuals with PD, making HRQoL an essential outcome measure in both clinical settings and research [5].

HRQoL instruments, particularly disease-specific measures, have become invaluable in capturing the personal impact of PD and the changes experienced by patients over time. Among these instruments, the Parkinson’s Disease Questionnaire-39 (PDQ-39) is the most widely used and validated tool for assessing HRQoL in patients with PD [5–8]. It covers eight dimensions of HRQoL: Activities of Daily Living, Bodily Discomfort, Cognitions, Communication, Emotional Well-Being, Mobility, Social Support, and Stigma, providing a comprehensive overview of the disease’s impact on HRQoL [9]. As a patient-reported outcome measure, the PDQ-39 reflects outcomes that matter to patients and aligns with regulatory guidance: both the European Medicines Agency and the U.S. Food and Drug Administration emphasize the use of validated instruments to inform benefit–risk assessment and, when appropriate, to support claims in product labeling [10, 11].

The PDQ-39 Summary Index, hereafter referred to as the Summary Index, is a composite score derived from the individual eight dimensions of the PDQ-39 and is frequently used in clinical trials as a primary outcome measure to evaluate the overall impact of PD on patients’ lives [12, 13]. While the Summary Index provides a valuable summary of HRQoL, it can obscure the nuances captured by the individual dimensions, each reflecting a distinct aspect of the disease’s impact. Understanding the relationship between these dimensions and clinical variables is crucial for tailoring interventions to address the specific challenges faced by patients with PD. However, this remains challenging due to the complex and multifaceted nature of the disease, where symptoms can vary widely among individuals. Notably, non-motor symptoms (NMS), which are often among the most disabling yet under-recognized aspects of PD, have been shown to considerably impact the quality of life [8]. The Non-Motor Symptoms Questionnaire (NMSQ) is a self-reported instrument of 30 yes/no items covering common non-motor symptoms in PD. The total NMSQ score is obtained by summing the number of symptoms the patient reports, yielding a score from 0 to 30, with higher scores reflecting a greater burden of non-motor symptoms. This simple sum provides a practical, validated measure of overall non-motor symptom burden in PD [14].

Traditional statistical models often fall short in capturing the nonlinear and high-dimensional relationships between the diverse clinical manifestations of PD and HRQoL. In response, ML methods have gained traction for their ability to model such complexity, with demonstrated success in predicting disease progression, motor subtypes, and treatment response [15–24]. While these advances highlight the potential of ML in neurodegenerative disease modeling, most applications to date have concentrated on motor symptoms, digital monitoring, and disease classification.

In contrast, ML models targeting HRQoL in PD, particularly using the PDQ-39, remain limited. Most existing studies treat HRQoL as a secondary outcome or focus solely on the global Summary Index, overlooking the predictive modeling of its individual dimensions [25–28]. Building on our earlier work published in the *Journal of Clinical Medicine* (2024), which focused on predicting the Summary Index, this study addresses a critical gap by extending the analysis to the eight individual PDQ-39 dimensions. By doing so, we aim to enhance the understanding of dimension-specific quality-of-life impacts and support more personalized clinical decision-making in PD care.

This study aims to achieve two critical objectives: first, to explore and clarify the relationship between standard clinical variables and both the PDQ-39 dimensions and the Summary Index; second, to develop and assess the potential of ML models for predicting these outcomes. By incorporating explainable ML techniques, this study seeks to enhance clinicians’ understanding of HRQoL outcomes, offering deeper insights into the factors most strongly associated with the different dimensions of HRQoL in PD. This approach is intended to inform more personalized treatment strategies, ultimately improving care and HRQoL for patients with PD.

## Materials and methods

### Study overview and analysis workflow

**Fig. 1:**
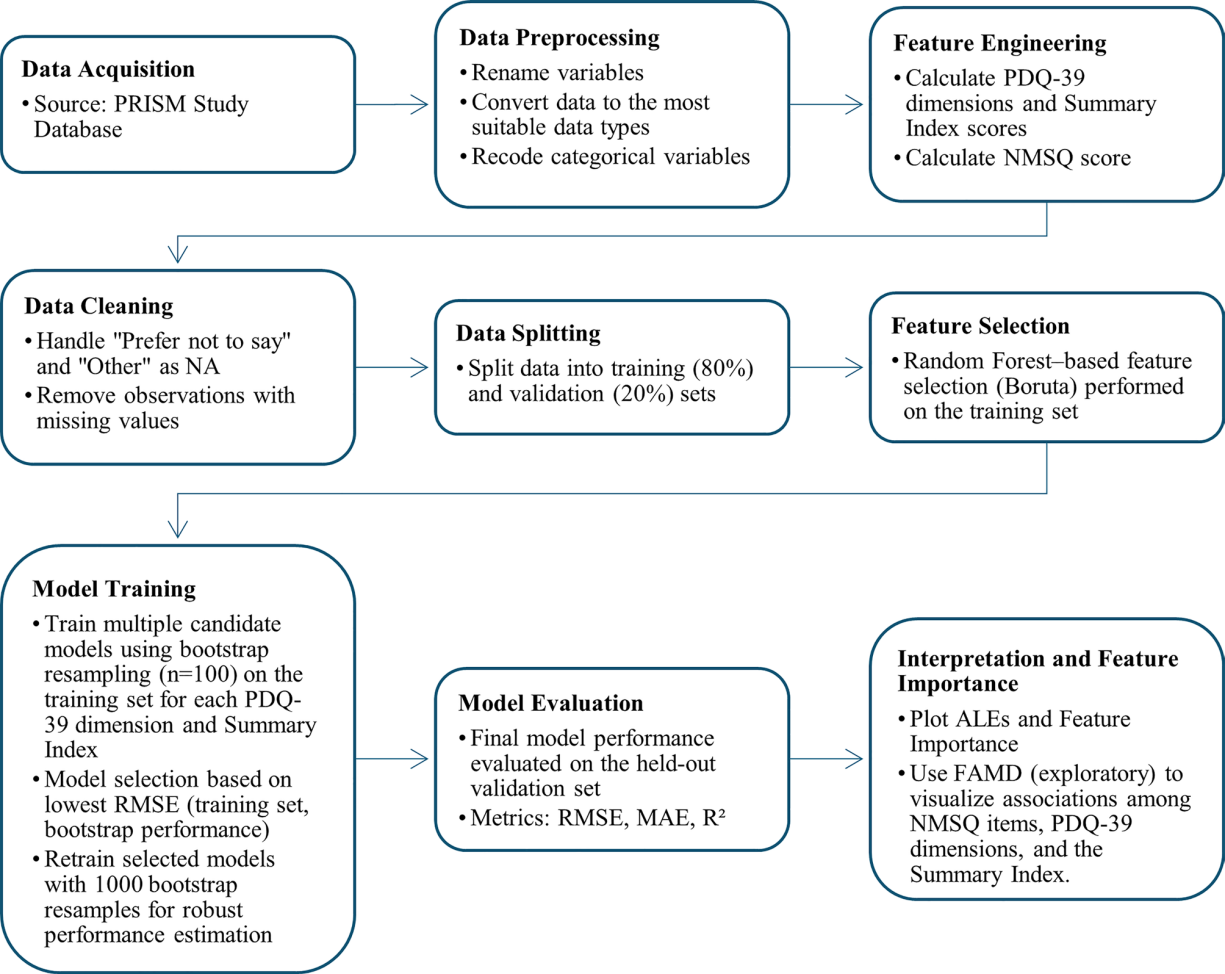
Diagram of the study’s workflow. PRISM, Parkinson’s Real-world Impact assesSMent; PDQ-39, Parkinson’s Disease Questionnaire-39; NMSQ, Non-Motor Symptoms Questionnaire; RMSE, Root Mean Squared Error; MAE, Mean Absolute Error; R², Coefficient of Determination; ALE, Accumulated Local Effects; FAMD, Factor Analysis of Mixed Data. Note: “Recode categorical variables” refers to converting categorical variables to suitable numeric/dummy-coded variables for modeling. Model selection was performed using the training set only; the validation set was reserved for final, unbiased performance assessment

Figure 1 summarizes the workflow from the PRISM data source and analytic sample selection through model development, evaluation, and interpretability. It is intended as an overview; full details are provided in the subsequent Methods subsections.

### Database

The data for this study were sourced from the Parkinson’s Real-world Impact assesSMent (PRISM) study, a cross-sectional observational research initiative conducted across six European countries: France, Germany, Italy, Portugal, Spain, and the United Kingdom. Detailed information on the study design and recruitment can be found in Tolosa et al., 2021 [29]. The PRISM study included 861 patients with PD and 256 caregivers, recruited via patient advocacy groups and clinical centers. Participants completed a comprehensive online survey comprising validated instruments, including the PDQ-39, the NMSQ, as well as questions on demographics, medication, comorbidities, disease characteristics, and healthcare resource utilization. This diverse database supports robust modeling and generalizability to the wider European PD population.

### Data preparation and analysis

The PRISM dataset underwent a data cleaning process using R statistical software (version 4.3.2) [30]. Subsequently, all analyses were performed with the same software to ensure consistency and reproducibility. For categorical variables such as gender, medication, and comorbidities, responses like “prefer not to say” and “other” were recoded as missing, as these options do not provide analyzable clinical information and would introduce noise if retained. Most variables in the dataset had low rates of missing data; however, a notable exception was observed for the NMSQ, which had 31.2% missing responses. This suggests a pattern of survey fatigue, where respondents’ engagement decreased as the questionnaire progressed, leading to an increased number of missing responses. This indicates that the missing data in the NMSQ likely follow a missing not at random (MNAR) pattern, suggesting an attrition effect in survey completion. Additionally, the variables “age at diagnosis” and “current medication” exhibited an MNAR pattern, likely due to patients not recalling their age at PD diagnosis or being unaware of their current medication.

Although we initially attempted to address missing values using Multivariate Imputation by Chained Equations (MICE), this approach resulted in models with worse predictive capabilities [31]. Therefore, only cases with complete data for all variables required for modeling were retained, resulting in a final analytic sample of 478 patients. No missing data remained in the analytic sample.

**Fig. 2:**
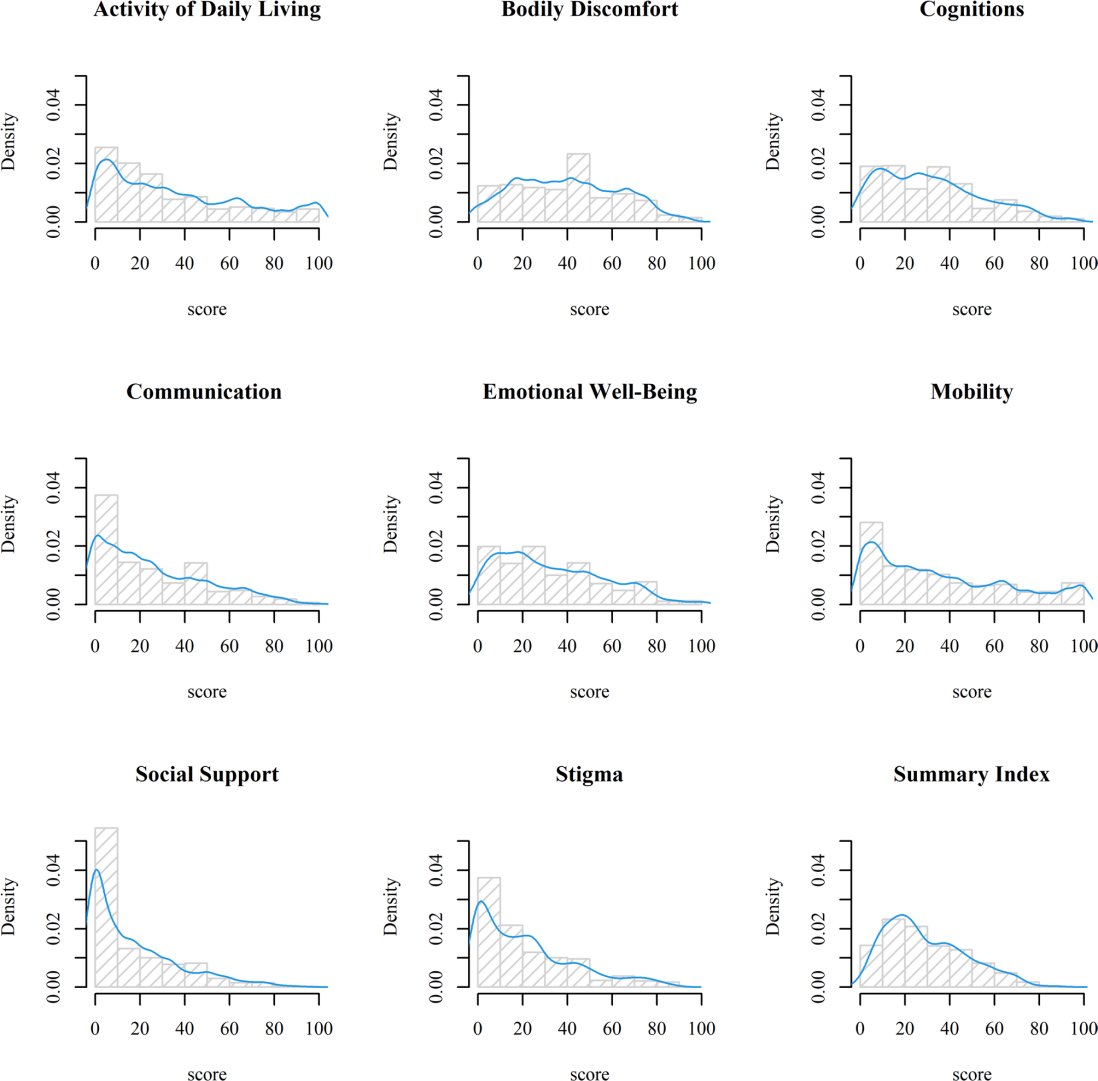
Distribution and density plots of PDQ-39 dimensions and Summary Index scores across 478 patients from the PRISM study

**Fig. 3:**
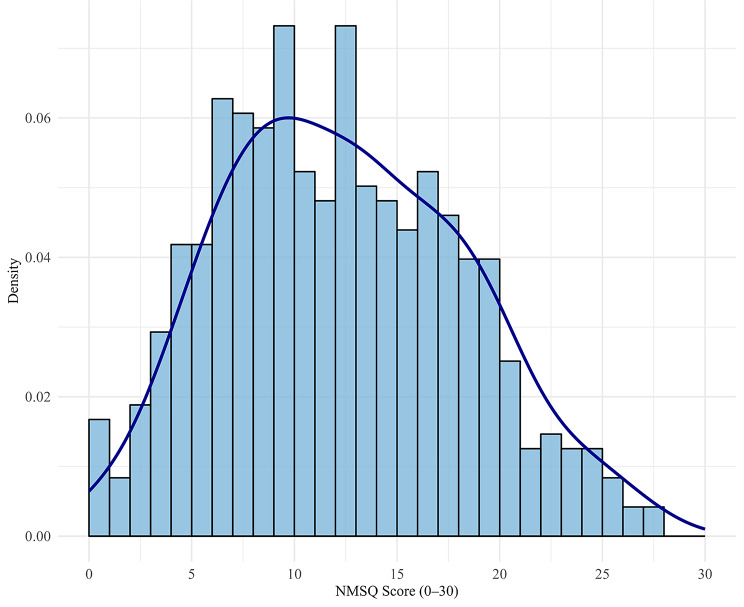
Distribution and density of NMSQ score across 478 patients from the PRISM study

The analytic sample (*n* = 478) comprised 46.9% female and 53.1% male participants, with a mean age of 65.6 years (Standard Deviation (SD) = 9.7 years). The distributions of the PDQ-39 dimensions and Summary Index are depicted in Fig. 2. The distribution of the NMSQ score is displayed in Fig. 3. Table 1 provides an overview of key variables from the 478 complete observations from PRISM database.

**Table 1:** Descriptive statistics of the complete observations from PRISM database

Characteristics	Overall (*N* = 478)
Demographics	
Age, years	
Mean (SD)	65.6 (9.7)
Median [IQR]	66.0 [59.0;72.0]
Min, Max	[38.0;91.0]
Gender	
Female	224 (46.9%)
Male	254 (53.1%)
Country	
France	19 (4.0%)
Germany	50 (10.5%)
Italy	141 (29.5%)
Portugal	45 (9.4%)
Spain	82 (17.2%)
UK	141 (29.5%)
Education	
University	206 (43.1%)
No University	272 (56.9%)
NMS Questionnaire	
Mean (SD)	12.5 (5.9)
Median [IQR]	12.0 [8.0;17.0]
Min, Max	[0.0;28.0]
Comorbidities	
Anxiety	72 (15.1%)
Asthma	25 (5.2%)
Cancer	23 (4.8%)
Chronic Obstructive Pulmonary Disease	10 (2.1%)
Dementia	24 (5.0%)
Depression	97 (20.3%)
Diabetes (type 1 or 2)	29 (6.1%)
Gastric ulcer	22 (4.6%)
Heart issues	36 (7.5%)
High blood pressure	126 (26.4%)
Kidney disease	9 (1.9%)
Liver disease	8 (1.7%)
Peripheral vascular arterial disease	18 (3.8%)
Rheumatic diseases	44 (9.2%)
Stroke	7 (1.5%)
PD Medication (current)	
Amantadine	47 (9.8%)
Anticholinergics	8 (1.7%)
Catechol-O-methyltransferase inhibitor	60 (12.6%)
Dopamine agonists	248 (51.9%)
Levodopa	404 (84.5%)
Monoamine oxidase-B inhibitor	191 (40.0%)
Rivastigmine	17 (3.6%)
PDQ-39	
Activities of Daily Living	
Mean (SD)	30.7 (27.1)
Median [IQR]	20.8 [8.3;45.8]
Min, Max	[0.0;100.0]
Bodily Discomfort	
Mean (SD)	39.9 (23.1)
Median [IQR]	41.7 [18.8;58.3]
Min, Max	[0.0;91.7]
Cognitions	
Mean (SD)	31.7 (22.6)
Median [IQR]	31.2 [12.5;43.8]
Min, Max	[0.0;93.8]
Communication	
Mean (SD)	25.7 (23.5)
Median [IQR]	16.7 [8.3;41.7]
Min, Max	[0.0;100.0]
Emotional Well-Being	
Mean (SD)	33.1 (23.5)
Median [IQR]	29.2 [12.5;50.0]
Min, Max	[0.0;100.0]
Mobility	
Mean (SD)	35.8 (29.9)
Median [IQR]	30.0 [10.0;57.5]
Min, Max	[0.0;100.0]
Social Support	
Mean (SD)	16.7 (19.7)
Median [IQR]	8.3 [0.0;25.0]
Min, Max	[0.0;91.7]
Stigma	
Mean (SD)	21.7 (21.4)
Median [IQR]	18.8 [6.2;31.2]
Min, Max	[0.0;87.5]
Summary Index	
Mean (SD)	29.4 (17.9)
Median [IQR]	25.1 [15.2;41.3]
Min, Max	[1.3;89.3]

IQR, interquartile range; NMS, Non-Motor Symptoms; PD, Parkinson’s disease; PDQ-39, Parkinson’s Disease Questionnaire-39; SD, standard deviation

Internal consistency of the PDQ-39 Summary Index and its eight dimensions was assessed using Cronbach’s α in the retained sample (*n* = 478). Scoring followed standard procedures, with all scores ranging from 0 to 100 and higher scores reflecting worse HRQoL. There were no missing items for the PDQ-39 in the analytic sample.

The dataset was then randomly partitioned into training (80%) and validation (20%) sets. This allocation allows for comprehensive model training on a diverse range of responses, critical for capturing the nuanced patterns of HRQoL assessments in PD, while still reserving a portion of the data for essential validation to assess model performance and overfitting.

### Model training

We selected machine learning approaches for their scalability and proven ability to capture complex, nonlinear relationships that are often missed by traditional statistical models. This framework supports robust prediction in moderate-sized datasets and can be readily extended to larger cohorts as more data becomes available.

For feature selection, we used the Boruta algorithm. This method is based on Random Forest models and works by checking which variables are truly useful for predicting each PDQ-39 dimension and the Summary Index [32–35]. Feature selection was performed exclusively on the training set after the initial 80/20 train/validation split, to avoid information leakage. Using this approach, we identified nine distinct sets of features specific to each of the PDQ-39 dimensions and the Summary Index (see Table 4 in the Results section). These nine sets were then utilized in training a diverse range of ML models, namely linear regression, boosted generalized linear model, extreme gradient boosting with linear models and decision trees, ridge regression, neural network regression, LASSO regression, random forest, k-nearest neighbors, support vector machines with radial basis function and polynomial kernels, and Gaussian processes (linear, polynomial, and radial kernels), incorporating essential pre-processing steps such as centering and scaling with the caret package [36].

To evaluate the performance and stability of these models in a dataset characterized by limited size and high variability, we utilized bootstrap resampling exclusively on the training set, using the standard nonparametric (Efron’s) bootstrap method [36, 37]. Bootstrap is a well-established approach that provides a more reliable estimate of the generalizability of machine learning models, particularly in smaller datasets, by generating multiple resampled sets and reducing the bias inherent in model evaluation [38]. Initially, each candidate model was evaluated using 100 bootstrap resamples, and the model with the lowest average Root Mean Squared Error (RMSE) in the training set was selected for each outcome, as RMSE is widely recognized for its effectiveness in assessing predictive performance by penalizing larger errors more than smaller ones [38]. The selected models were then retrained on the training set using 1000 bootstrap resamples to obtain robust estimates of their predictive performance.

The held-out validation set (20% of the data) was reserved strictly for final, unbiased model evaluation and was not subjected to bootstrap resampling. Validation set metrics were calculated as single-point estimates to reflect true out-of-sample predictive performance.

### Model evaluation

The performance of the models was evaluated using multiple metrics on both the training set (80%) and the validation set (20%) to assess generalizability and overfitting. The validation set was specifically reserved for the final evaluation to ensure an unbiased assessment of models’ performance. Mean Absolute Error (MAE) quantifies the average magnitude of prediction errors, providing a straightforward measure of how much predicted values deviate from true values. RMSE offers insight into the typical magnitude of prediction errors by emphasizing larger errors due to its squared nature, while the Coefficient of Determination (R²) indicates the proportion of variance in the dependent variable explained by the model, highlighting its explanatory power. In addition to these metrics, scatter plots of predicted versus true values were used to evaluate how well predictions matched the observations, and studentized residual plots were examined for patterns or systematic errors. Together, these quantitative and visual evaluation tools provide a comprehensive assessment of model performance and robustness.

Because the Summary Index captures overall HRQoL and each model targets different aspects of patient experience, we evaluated and compared the predictive performance across all models. This allows us to see whether overall or specific outcomes are predicted with lower error and gives a broader view of which aspects of HRQoL are most predictable in this dataset.

### Interpretable machine learning analysis

To elucidate the importance and model reliance on each feature within our predictive models, we employed interpretable ML techniques, with a particular focus on Permutation Feature Importance (PFI). Utilizing the iml package in R, PFI provides deeper insight into the behavior of the ML models [39]. The core concept of PFI is to measure the increase in the model’s prediction error after the permutation of each feature’s values, which disrupts the relationship between the feature and the true outcome. A considerable increase in error indicates that the model depends heavily on that feature for predictions, while minimal or no change implies that the feature is not as relevant in the predictive process [40]. However, it’s crucial to recognize that even features with minimal changes in prediction error may still contribute meaningfully to the overall performance of the model. These variables, while not strongly predictive on their own, can add complementary information and interact with other predictors, which may enhance model generalizability [41]. Feature importance was estimated using the final fitted model (i.e., the best-performing algorithm for each dimension and the Summary Index) applied to the independent validation set. We assessed feature importance in our models using MAE as the error metric for PFI calculations. This process was repeated 100 times to ensure robust estimates, with the results averaged to provide a clearer view of the models’ reliance on each variable.

Additionally, to understand the impact of specific variables on our outcomes, we employed Accumulated Local Effects (ALE) plots. ALE plots allow us to visualize the effect of a feature on the predicted outcome while accounting for interactions with other features, particularly in datasets with correlated predictors [40, 42]. By generating ALE plots for the most relevant features identified through PFI, we were able to interpret how changes in these features affect the PDQ-39 dimensions and the Summary Index.

Furthermore, during our analysis, we observed that the NMSQ score (sum of non-motor symptoms reported by the patient) consistently ranked as the most important predictor across all ML models, when compared to the full set of clinical and demographic variables included in the models (see Table 4 in the Results section).

### Exploratory multivariate analysis using FAMD

Given that the NMSQ comprises 30 items assessing various symptoms, we performed a Factor Analysis of Mixed Data (FAMD) to explore the underlying structure and relationships among these items, the eight PDQ-39 dimensions, and the Summary Index. FAMD is particularly suited for mixed data types and simplifies data complexity by identifying principal dimensions that capture the most variance (inertia) in the dataset [43]. This approach elucidates how variables co-vary by grouping associated variables in proximity, thereby revealing underlying patterns and dimensions. FAMD is exploratory and should not be interpreted as indicating predictive importance or causality.

## Results

### Psychometric Properties of the PDQ-39

Internal consistency of the PDQ-39 Summary Index and its eight dimensions was assessed using Cronbach’s 𝛼. Reliability was excellent for the Summary Index (α = 0.96) and generally high for individual dimensions, ranging from α = 0.71 (Bodily Discomfort) to α = 0.96 (Mobility). Detailed results for all dimensions are presented in Table 2.

**Table 2:** Internal consistency (Cronbach’s 𝛼) for the PDQ-39 summary index and individual dimensions (0–100 scale, *n* = 478)

Dimensions	Cronbach 𝛼
Activities of Daily Living	0.91
Bodily Discomfort	0.71
Cognitions	0.77
Communication	0.83
Emotional Well-Being	0.90
Mobility	0.96
Social Support	0.73
Stigma	0.76
Summary Index	0.96

Cronbach’s α ≥ 0.70 indicates acceptable internal consistency; α ≥ 0.80 indicates good consistency; α ≥ 0.90 indicates excellent consistency

### Predictive model performance

Performance metrics for the training set are reported as mean (95% CI) [SD] from 1000 bootstrap resamples; validation set values are single point estimates from the independent validation set. Table 3 presents a summary of the performance metrics (MAE, RMSE, and R²) for both training and validation sets, alongside the selected models for each PDQ-39 dimension and the Summary Index. The Extreme Gradient Boosting with Decision Trees (xgbTree) model was selected as the best-performing model for both the Summary Index and the Activities of Daily Living dimension, while the Gaussian Process with Polynomial Kernel (gaussprPoly) was chosen for all other dimensions.

**Table 3:** Results

PDQ-39 dimension / Summary Index	Training			Validation			Model selected
	MAE	RMSE	*R*²	MAE	RMSE	*R*²	
Activities of Daily Living	15.12 (13.52–16.98) [0.89]	19.40 (17.21–21.80) [1.14]	0.47 (0.35–0.57) [0.06]	18.33	24.41	0.33	xgbTree
Bodily Discomfort	15.27 (13.79–16.78) [0.77]	18.90 (17.09–20.63) [0.91]	0.29 (0.18–0.40) [0.05]	17.82	21.63	0.32	gaussprPoly
Cognitions	12.55 (11.29–13.88) [0.67]	15.64 (13.96–17.14) [0.82]	0.52 (0.41–0.61) [0.05]	12.39	16.20	0.53	gaussprPoly
Communication	14.89 (13.34–16.41) [0.80]	18.88 (16.83–20.91) [1.07]	0.36 (0.25–0.47) [0.06]	13.70	18.44	0.37	gaussprPoly
Emotional Well-Being	13.45 (12.08–14.83) [0.74]	17.24 (15.49–18.99) [0.91]	0.46 (0.36–0.56) [0.05]	14.78	19.50	0.36	gaussprPoly
Mobility	17.96 (16.30-19.64) [0.87]	22.14 (19.99–24.31) [1.12]	0.44 (0.33–0.54) [0.05]	18.02	22.88	0.48	gaussprPoly
Social Support	13.50 (12.16–14.90) [0.70]	17.44 (15.49–19.41) [1.04]	0.17 (0.08–0.28) [0.05]	14.87	19.08	0.26	gaussprPoly
Stigma	15.49 (14.00-17.02) [0.80]	19.40 (17.34–21.54) [1.08]	0.20 (0.09–0.30) [0.05]	14.96	19.34	0.23	gaussprPoly
Summary Index	8.99 (8.04–9.91) [0.49]	11.45 (10.16–12.73) [0.64]	0.58 (0.48–0.67) [0.05]	9.60	12.66	0.56	xgbTree

Values for the training set are reported as mean (95% CI) [SD] from 1000 bootstrap resamples. Validation set metrics are single-point estimates computed on the independent validation set. All model predictors were standardized (centered and scaled) using caret’s preprocessing step prior to training. MAE, Mean Absolute Error; RMSE, Root Mean Squared Error; R², Coefficient of Determination

Training and validation results were broadly similar across outcomes, indicating limited overfitting. The Summary Index model outperformed the individual dimensions models, achieving the lowest MAE (9.60) and RMSE (12.66), and the highest R² (0.56) in the validation set. The Cognitions dimension model also demonstrated strong predictive performance, with an R² of 0.53, MAE of 12.39, and RMSE of 16.20 in the validation set. This indicates that the model explained over half of the variance in Cognitions scores and produced relatively low average prediction errors, suggesting good predictive performance for this dimension.

The Mobility model explained nearly half the variance (R²=0.48) but had relatively high MAE/RMSE (18.02/22.88), suggesting substantial average prediction errors. This indicates that while the model captures general trends in Mobility, its predictions for individual patients may lack precision due to larger errors. The Emotional Well-Being model had an R² of 0.36, with an MAE of 14.78 and RMSE of 19.50. Although the R² is lower than that of the Mobility model, the lower MAE and RMSE values indicate smaller average prediction errors, reflecting moderate predictive capability with better precision compared to the Mobility model.

Interestingly, the models for Stigma and Social Support, despite having lower R² values (0.23 and 0.26, respectively), exhibited lower MAE and RMSE values compared to models like Mobility. The Stigma model had an MAE of 14.96 and RMSE of 19.34, while the Social Support model had an MAE of 14.87 and RMSE of 19.08. These lower error metrics suggest that, on average, the predictions for Stigma and Social Support were closer to the true values, even though the models explained less variance in the outcomes. This could be attributed to lower variability in the Stigma and Social Support scores, where even small prediction errors can result in lower R² values due to the limited variance in these dimensions.

The Activities of Daily Living and Bodily Discomfort models showed lower predictive performance. The Activities of Daily Living model had an R² of 0.33, with the highest MAE (18.33) and RMSE (24.41), indicating considerable prediction errors. Similarly, the Bodily Discomfort model had an R² of 0.32, an MAE of 17.82, and an RMSE of 21.63. These higher error metrics suggest that these dimensions were more challenging to predict, likely reflecting variability from factors not captured by the available features.

### Scatter and residuals analysis

**Fig. 4:**
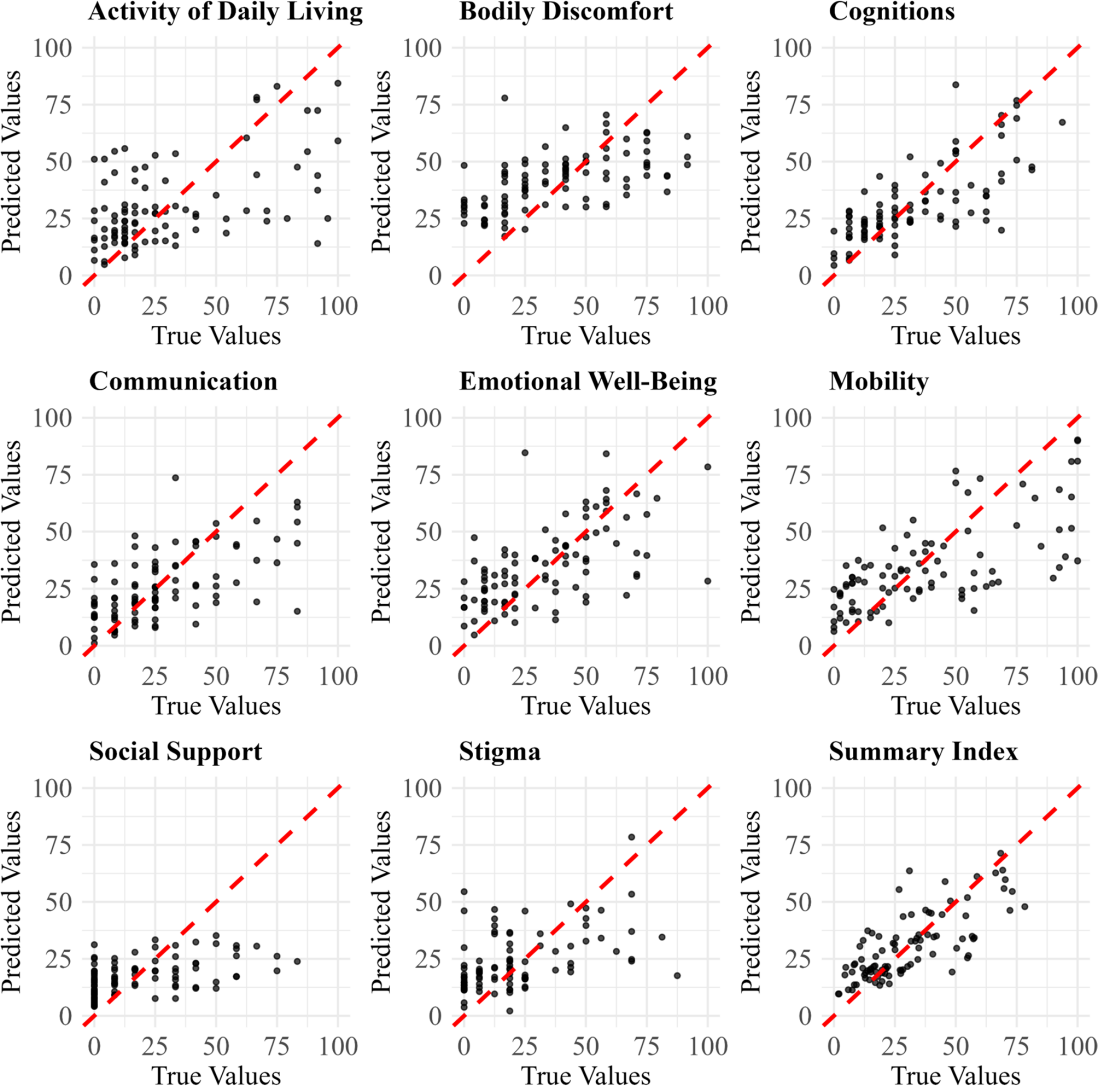
Predicted vs. true values for PDQ-39 dimensions and summary index. The black dots represent individual predictions, and the red dashed line represents the line of perfect prediction where predicted values would equal true values. Predictions were generated on the independent validation set using the following fitted algorithms: xgbTree for Summary Index and Activities of Daily Living; and gaussprPoly for all other dimensions

In Fig. 4, which presents scatter plots of predicted versus true values, the Summary Index model demonstrates the best fit, with predictions closely clustering around the red dashed line, reflecting its superior performance metrics. Cognitions, Emotional Well-Being, and Communication also show an adequate fit, with reasonable alignment between predicted and true values. However, for Mobility, while the model performs well for lower true values, it tends to underpredict higher scores. Similarly, the models for Bodily Discomfort, Social Support, and Stigma slightly overpredict lower values and underpredict higher ones, indicating a regression toward the mean. The Activities of Daily Living model emerges as having the highest prediction error, with substantial deviations from the red dashed line, signaling lower predictive performance.

**Fig. 5:**
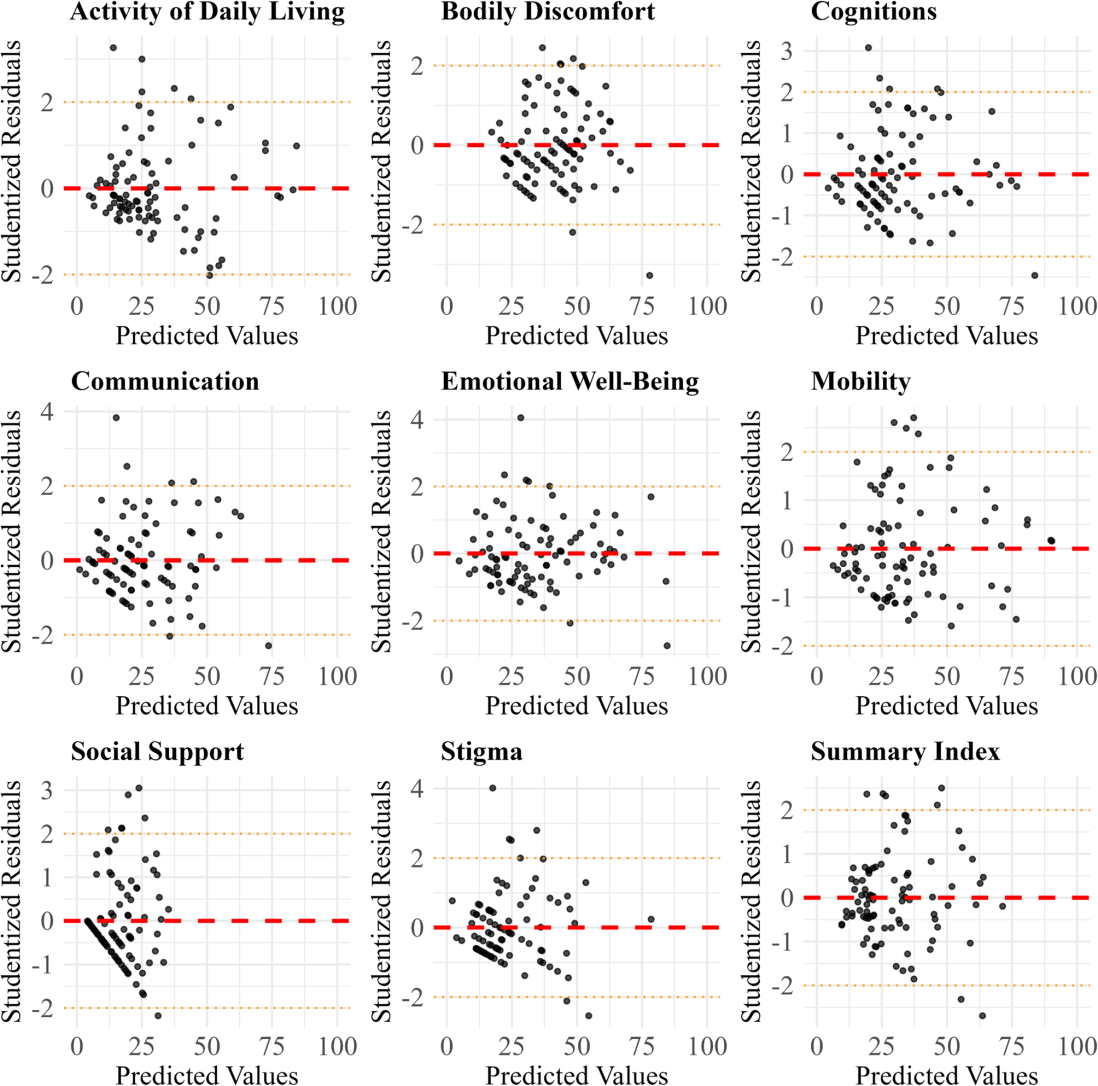
Studentized residuals for each PDQ-39 dimension and summary index model, plotted against predicted values from the validation set. The red dashed line indicates zero residual error. The orange dotted lines mark ± 2 standard deviations. Residuals were generated on the independent validation set using the following fitted algorithms: xgbTree for Summary Index and Activities of Daily Living; and gaussprPoly for all other dimensions

In Fig. 5, which presents residual plots for each model, we observe distinct patterns in model performance. The residuals for the Summary Index are evenly distributed around zero with low spread, reflecting a well-fitted model with strong predictive performance. Similarly, the Cognitions and Emotional Well-Being models show residuals that are clustered around zero, indicating a solid fit and minimal error variance. Both Communication and Bodily Discomfort exhibit a wider spread of residuals from zero, although their distribution remains centered, indicating moderate performance. While these models capture some trends in the data, their larger residual spread suggests less precision compared to models like the Summary Index and Cognitions. The Mobility model demonstrates a good fit for lower predicted values, but exhibits an asymmetrical spread at higher values, with a greater number of residuals above zero, suggesting underprediction for larger outcomes. The models for Activities of Daily Living and Stigma exhibit larger residuals across the prediction spectrum. In particular, Activities of Daily Living shows consistently large errors, indicating poor performance, while Stigma and Social Support display heteroscedasticity, with increasing residual variance as predicted values rise, indicating that the models become less reliable when predicting higher values.

Overall, the scatter and residual analyses confirm varying model performance: Summary Index, Cognitions, and Emotional Well-Being demonstrate strong predictive capabilities. Communication and Bodily Discomfort show moderate performance, and Activities of Daily Living exhibits the poorest performance. Four models display specific patterns: Communication, Social Support, and Stigma exhibit heteroscedasticity, while Mobility shows asymmetric residual distribution.

### Feature importance

**Fig. 6:**
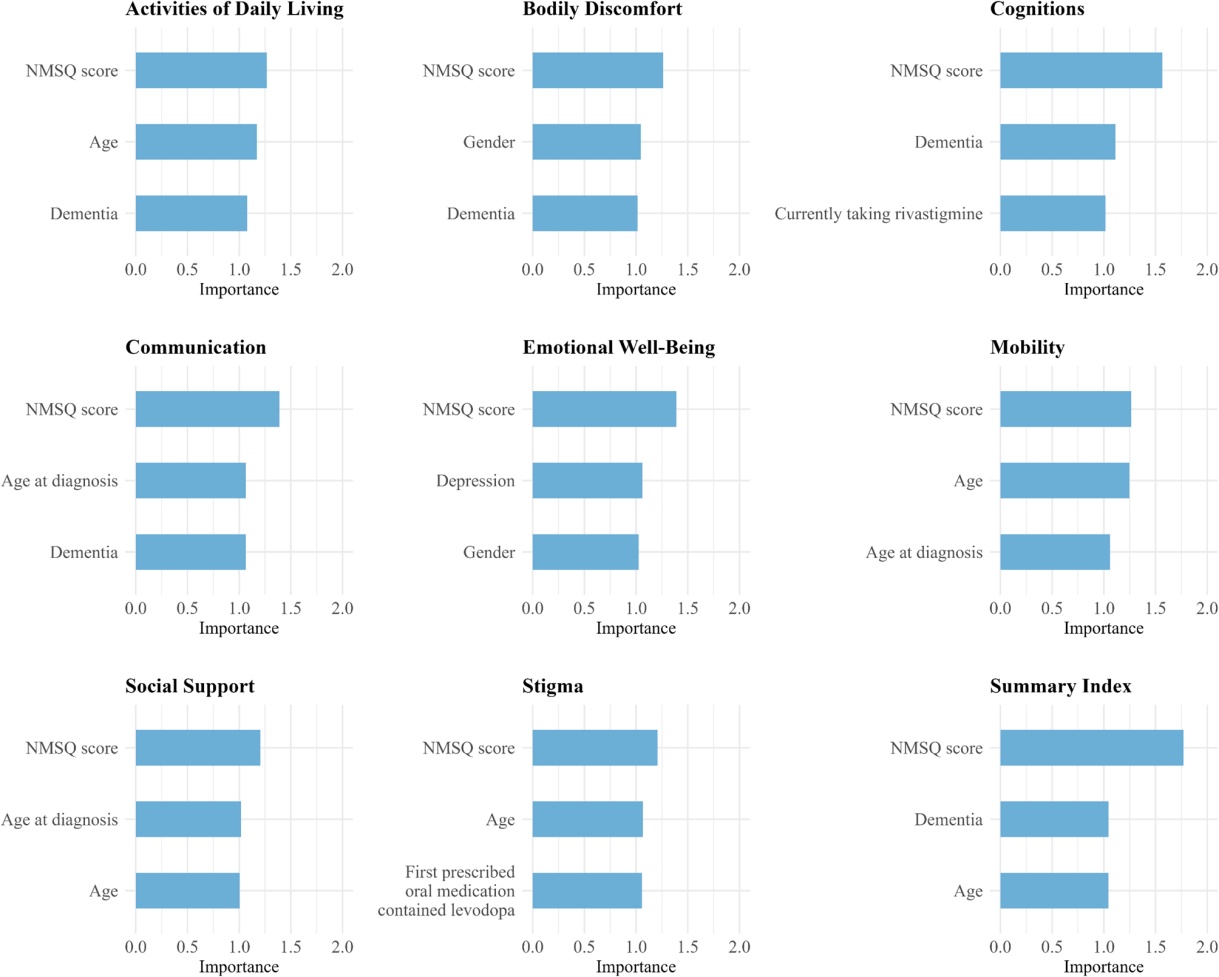
Feature importance for predictive models of PDQ-39 dimensions and summary index. Importance was assessed as the mean increase in MAE after permuting each feature (100 permutations), using the final fitted models applied to the independent validation set

In Fig. 6, the feature importance analysis highlights the universal relevance of the NMSQ score across all models, consistently ranking as the top predictor in every PDQ-39 dimension and the Summary Index. These findings reinforce the central predictive role of NMSQ and align with prior evidence that greater non-motor symptom burden is highly associated with poorer HRQoL [25, 26, 44]. Other clinical variables also showed importance in specific dimensions. Age was particularly relevant in both Mobility and Activities of Daily Living. Notably, for Mobility, age had nearly the same importance as the NMSQ score, indicating its critical role in predicting outcomes in this dimension. Dementia emerged as an important predictor in the Cognitions dimension, aligning with the well-established link between cognitive decline and dementia in patients with PD. Despite these findings, the overall predictive contribution of these clinical variables was less pronounced than that of the NMSQ score. Feature importance values for all selected variables are displayed in Table 4.

**Table 4:** Features selected by Boruta algorithm and their permutation-based importance in each PDQ-39 dimension and summary index model

Variables	Importance	Permutation error
Activities of Daily Living model		
NMSQ score	1.27	23.21
Age	1.17	21.47
Dementia	1.08	19.74
Age at diagnosis	1.05	19.27
Currently taking Monoamine oxidase-B inhibitor	1.02	18.61
Cancer	1.01	18.45
Number of comorbidities	1.00	18.33
Peripheral vascular arterial disease	1.00	18.33
Gastric ulcer	1.00	18.33
Anxiety	1.00	18.29
Currently taking rivastigmine	1.00	18.26
Gender	0.99	18.18
Country	0.98	18.00
Bodily Discomfort model		
NMSQ score	1.26	22.48
Gender	1.05	18.62
Dementia	1.01	18.06
Number of comorbidities	1.00	17.90
First prescribed oral medication contained levodopa	1.00	17.85
Age at diagnosis	1.00	17.84
Age	1.00	17.83
Cancer	1.00	17.78
Anxiety	1.00	17.76
Cognitions model		
NMSQ score	1.57	19.39
Dementia	1.11	13.78
Currently taking rivastigmine	1.02	12.58
Age	1.02	12.58
Country	1.01	12.50
Depression	1.01	12.48
Heart issues	1.01	12.46
Gastric ulcer	1.01	12.46
Peripheral vascular arterial disease	1.01	12.46
Age at diagnosis	1.00	12.44
Number of comorbidities	1.00	12.44
First prescribed oral medication contained levodopa	1.00	12.40
Communication model		
NMSQ score	1.39	19.04
Age at diagnosis	1.06	14.59
Dementia	1.06	14.57
Age	1.02	13.95
Country	1.01	13.89
Depression	1.00	13.71
Anxiety	1.00	13.69
Currently taking rivastigmine	1.00	13.65
Currently taking Monoamine oxidase B inhibitor	0.99	13.61
First prescribed oral medication contained levodopa	0.99	13.61
Cancer	0.99	13.59
Number of comorbidities	0.99	13.56
Emotional Well-Being model		
NMSQ score	1.39	20.53
Depression	1.06	15.70
Gender	1.03	15.16
Number of comorbidities	1.02	15.03
Anxiety	1.01	14.99
Country	1.01	14.96
Dementia	1.00	14.85
Age	1.00	14.74
Peripheral vascular arterial disease	0.99	14.60
Age at diagnosis	0.98	14.47
Mobility model		
NMSQ score	1.27	22.80
Age	1.25	22.48
Age at diagnosis	1.06	19.09
Dementia	1.05	18.91
First prescribed oral medication contained levodopa	1.02	18.42
Currently taking rivastigmine	1.01	18.29
Depression	1.01	18.27
Peripheral vascular arterial disease	1.01	18.21
Currently taking Levodopa	1.01	18.12
Anxiety	1.00	18.09
Rheumatic diseases	1.00	17.99
Number of comorbidities	1.00	17.97
Social Support model		
NMSQ score	1.21	17.93
Age at diagnosis	1.02	15.12
Age	1.01	14.95
Anxiety	1.00	14.92
Number of comorbidities	1.00	14.89
Gastric ulcer	1.00	14.87
Depression	1.00	14.87
Stigma model		
NMSQ score	1.21	18.07
Age	1.07	15.96
First prescribed oral medication contained levodopa	1.06	15.82
Currently taking rivastigmine	1.04	15.62
Anxiety	1.04	15.55
Kidney disease	1.04	15.49
Age at diagnosis	1.01	15.14
Summary Index model		
NMSQ score	1.77	17.00
Dementia	1.04	10.03
Age	1.04	10.02
Age at diagnosis	1.04	9.94
Cancer	1.01	9.69
Depression	1.00	9.62
Peripheral vascular arterial disease	1.00	9.62
Anxiety	1.00	9.60
Gastric ulcer	1.00	9.60
Currently taking rivastigmine	1.00	9.60
Country	1.00	9.59
Number of comorbidities	1.00	9.59

Features selected through Boruta for each of the models. Feature Permutation Importance: Mean increase in MAE after permuting the feature (100 permutations); permutation error: standard deviation of the MAE increase across permutations

### Accumulated local effects

**Fig. 7:**
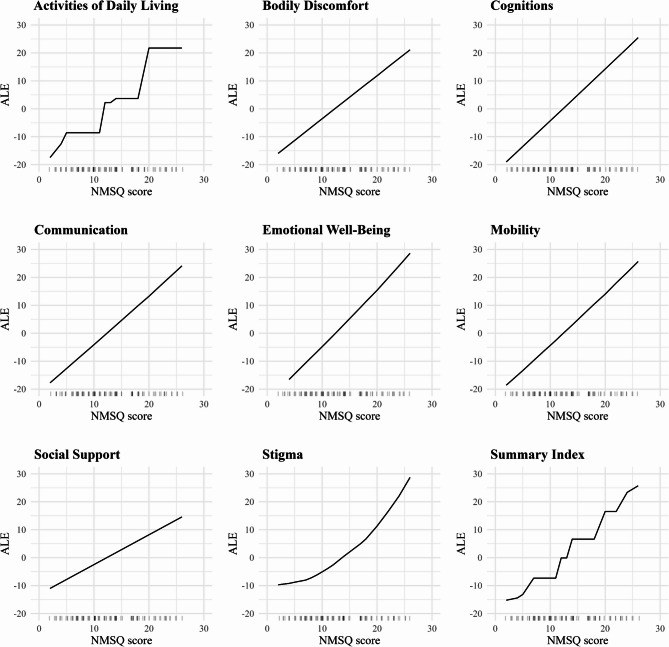
ALE plots of NMSQ scores on PDQ-39 dimensions and Summary Index, computed from the final fitted models and evaluated on the independent validation set

The ALE plots in Fig. 7 illustrate the effect of increasing NMSQ scores on the predicted values for each dimension and the Summary Index. ALE plots allow us to visualize the isolated effect of a single feature, in this case, the NMSQ scores, on the models’ predictions while accounting for interactions with other features. The x-axis represents the NMSQ score values, and the y-axis represents the accumulated local effect of these scores on the predicted outcomes, centered around zero, where zero indicates no impact on the predicted outcome. An upward trend in ALE values indicates that as NMSQ scores increase, the model predicts worsening HRQoL outcomes, reflecting the detrimental impact of non-motor symptoms.

In general, higher NMSQ scores were associated with worsening HRQoL, as evidenced by increasing ALE values across dimensions. However, the pattern and strength of this relationship varied by dimension. Both the Activities of Daily Living dimension and the Summary Index displayed pronounced stepwise patterns in the ALE plots, which is consistent with the behavior of the tree-based extreme gradient boosting models (xgbTree) selected for these two outcomes. These models create plateaus and jumps in predictions, which appear as steps in the ALE curves. The discrete NMSQ score scale (0–30) may further accentuate this appearance. In contrast, the Stigma dimension showed a steep increase at higher NMSQ scores, highlighting the pronounced impact of non-motor symptoms on patients’ perceived social standing and experiences of stigma. For the remaining dimensions, the relationship between NMSQ scores and predicted outcomes appeared fairly linear, indicating a consistent association between higher NMSQ scores and worse predicted HRQoL in these dimensions.

### Factor analysis of mixed data

**Fig. 8:**
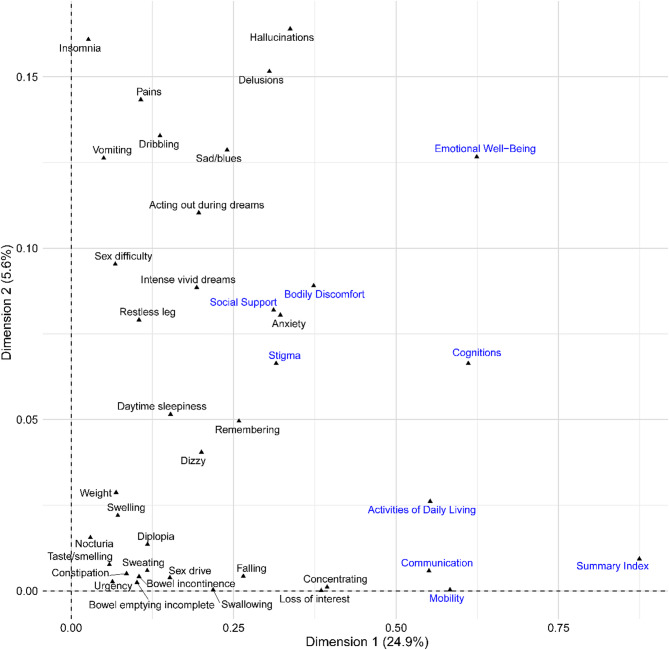
FAMD of NMSQ Items, PDQ-39 dimensions, and Summary Index. PDQ-39 dimensions and summary index are colored in blue. Axis percentages show the share of total inertia explained (Dim1 24.9%, Dim2 5.6%; 30.5% combined). Points that are closer indicate stronger associations; this is exploratory and not an evidence of causality or predictive importance

In Fig. 8, the FAMD plot visualizes associations in a low-dimensional factor space: closer proximity indicates stronger associations (co-variation), whereas greater distances reflect weaker associations. Summary Index, Cognitions, and Emotional Well-Being are the most distant from other variables. Their isolation may reflect broader, non-specific associations with multiple NMS, rather than ties to particular items. Activities of Daily Living, Communication, and Mobility appear relatively close to each other. Although they remain distant from individual NMS, their proximity suggests some level of association (co-variation) among these dimensions. This suggests that patients’ daily functioning, communication, and mobility may reflect shared underlying factors, rather than specific non-motor symptoms. Social Support, Bodily Discomfort, and Stigma form a cluster near Anxiety, indicating an association. In particular, Social Support is positioned very close to Anxiety, indicating a strong association between perceived social support and anxiety levels in patients with PD.

## Discussion

### Principal results

Our study demonstrates that while the Summary Index and the Cognitions dimension can be predicted with relatively low prediction error using common clinical variables and NMS, other dimensions present more challenges. Models for these two areas showed strong performance, indicating that overall HRQoL and cognitive aspects can be effectively estimated. In contrast, dimensions such as Mobility, Activities of Daily Living, and Bodily Discomfort were more difficult to predict, suggesting that additional variables may be necessary to improve model fit for these aspects of HRQoL.

The NMSQ score emerged as the most important feature across all ML models, underscoring the pervasive association of NMS with HRQoL in patients with PD. This aligns with previous studies highlighting the substantial burden of NMS on patients’ daily lives and emphasizes the need for comprehensive management strategies addressing both motor and non-motor symptoms in PD [25, 26, 44]. Apart from the NMSQ score, certain clinical variables were important predictors for specific dimensions. Age was notably important for Mobility and Activities of Daily Living, reflecting the impact of age-related decline on functional abilities. Dementia considerably contributed to the prediction of the Cognitions dimension, aligning with established associations between cognitive decline and dementia in PD [45].

The ALE plots revealed that higher NMSQ scores are associated with worsening HRQoL across all dimensions, with some exhibiting nonlinear relationships. Specifically, the Stigma dimension’s predicted scores increased nonlinearly, with steeper rises at higher NMSQ scores. This indicates that as the burden of NMS becomes more severe, feelings of stigma disproportionately intensify, highlighting the escalating impact of NMS on this aspect of HRQoL.

Furthermore, the FAMD visualization offered a nuanced understanding of the associations between NMS, PDQ-39 dimensions, and the Summary Index. Summary Index, Cognitions, and Emotional Well-Being were positioned distantly from other variables, suggesting broader, non-specific associations with multiple NMS rather than ties to particular symptoms. In contrast, Social Support, Bodily Discomfort, and Stigma clustered near Anxiety, indicating an association. This suggests that targeted interventions addressing specific NMS may benefit certain HRQoL dimensions.

Overall, these findings reflect the multifaceted nature of HRQoL in PD. The varying model performance suggests that incorporating additional variables may be necessary to improve prediction for certain dimensions of HRQoL.

### Comparison to previous work

Our findings corroborate prior research that emphasizes the considerable impact of NMS on HRQoL in patients with PD [25, 26, 44]. By employing ML models to predict individual PDQ-39 dimensions, our study extends previous work that predominantly focused on overall HRQoL. This approach provides a more detailed understanding of how specific symptoms and clinical variables are associated with distinct aspects of patients’ lives. Moreover, we addressed potential overfitting issues identified in our earlier study [27] by refining our modeling techniques and including additional variables, resulting in more robust and generalizable models. The use of interpretable ML methods offers novel insights into the complex relationships between NMS and HRQoL dimensions, contributing to a deeper comprehension of factors affecting patient well-being.

### Limitations

While our study provides important insights, a couple of limitations should be acknowledged. First, there was a considerable amount of missing data, particularly towards the end of the database, suggesting that participants experienced survey fatigue and stopped completing the survey. We attempted data imputation to address the missing values, but this resulted in worse model performance. Consequently, we excluded incomplete observations, resulting in a reduced sample size, which may have affected the generalizability and robustness of our models. Second, the models were developed using data from European patients, which may limit their applicability to other populations.

### Future directions

Future studies should aim to include more diverse populations from different demographic and cultural backgrounds to enhance the generalizability of the models. Incorporating psychosocial factors such as socioeconomic status, social support networks, and environmental influences may reduce prediction error for HRQoL dimensions like Stigma and Social Support. Additionally, using larger datasets or exploring methods to effectively handle missing data could enhance predictions for challenging dimensions. Longitudinal studies would also be beneficial to model disease progression and capture changes in HRQoL over time.

## Conclusions

Our study confirms that NMS are critical predictors of HRQoL in patients with PD. Utilizing ML models, we successfully predicted the Summary Index and the Cognitions dimension with relatively low prediction error, underscoring the profound impact of NMS on both overall and cognitive aspects of HRQoL. However, predicting other dimensions such as Mobility, Activities of Daily Living, and Bodily Discomfort remains challenging, indicating the need for additional variables to enhance predictive performance. The crucial role of NMS for predicting HRQoL emphasizes the necessity for integrated management approaches that address both motor and non-motor symptoms in PD.

Despite limitations related to missing data and the study’s European-centric dataset, our findings offer valuable insights into the complex interplay between NMS and HRQoL. By identifying specific clinical variables associated with certain HRQoL dimensions, our study contributes to the development of more targeted and effective interventions aimed at improving patient outcomes. Future research should focus on incorporating diverse populations, psychosocial factors, and longitudinal data to further refine predictive models and enhance HRQoL outcomes for patients with PD.

In conclusion, this study advances our understanding of how NMS affect various dimensions of HRQoL in PD, demonstrating the potential of ML models in personalizing patient care and informing clinical decision-making. These insights pave the way for more nuanced approaches to managing PD, ultimately aiming to improve the quality of life for those affected by this debilitating disease.

## Data Availability

The data used in this study were obtained from the Parkinson’s Real-world Impact Assessment (PRISM) database (https://next.prism.bial.com/), accessed on 18 February 2022.

## Abbreviations

ALE: Accumulated Local Effects
CI: Confidence Interval
FAMD: Factor Analysis of Mixed Data
gaussprPoly: Gaussian Process with Polynomial Kernel
HRQoL: Health—Related Quality of Life
IQR: Interquartile Range
MAE: Mean Absolute Error
MICE: Multivariate Imputation by Chained Equations
ML: Machine Learning
MNAR: Missing Not At Random
NMS: Non—Motor Symptoms
NMSQ: Non—Motor Symptoms Questionnaire
PD: Parkinson’s Disease
PDQ: 39—Parkinson’s Disease Questionnaire—39
PRISM: Parkinson’s Real—world Impact Assessment
R²: Coefficient of Determination
RMSE: Root Mean Squared Error
SD: Standard Deviation
xgbTree: Extreme Gradient Boosting with Decision Trees
